# *schema*: an open-source, distributed mobile platform for deploying mHealth research tools and interventions

**DOI:** 10.1186/s12874-020-00973-5

**Published:** 2020-04-25

**Authors:** Adrian B. R. Shatte, Samantha J. Teague

**Affiliations:** 1 School of Science, Engineering and Information Technology, Federation University, 100 Clyde Rd, Berwick, Melbourne, VIC 3806 Australia; 2 Centre for Social and Early Emotional Development, School of Psychology, Faculty of Health, Deakin University, Geelong, Australia

**Keywords:** mHealth, Ecological momentary assessment, Experience sampling, App-based intervention

## Abstract

**Background:**

Mobile applications for health, also known as ‘mHealth apps’, have experienced increasing popularity over the past ten years. However, most publicly available mHealth apps are not clinically validated, and many do not utilise evidence-based strategies. Health researchers wishing to develop and evaluate mHealth apps may be impeded by cost and technical skillset barriers. As traditionally lab-based methods are translated onto mobile platforms, robust and accessible tools are needed to enable the development of quality, evidence-based programs by clinical experts.

**Results:**

This paper introduces *schema*, an open-source, distributed, app-based platform for researchers to deploy behavior monitoring and health interventions onto mobile devices. The architecture and design features of the platform are discussed, including flexible scheduling, randomisation, a wide variety of survey and media elements, and distributed storage of data. The platform supports a range of research designs, including cross-sectional surveys, ecological momentary assessment, randomised controlled trials, and micro-randomised just-in-time adaptive interventions. Use cases for both researchers and participants are considered to demonstrate the flexibility and usefulness of the platform for mHealth research.

**Conclusions:**

The paper concludes by considering the strengths and limitations of the platform, and a call for support from the research community in areas of technical development and evaluation. To get started with *schema*, please visit the GitHub repository: https://github.com/schema-app/schema.

## Background

Mobile applications for health, also known as mHealth apps, have become increasingly popular with researchers, clinicians, and the public as effective tools for monitoring and improving health behaviors. Recent reports indicate that there are now over 318,000 mHealth apps available across the Android and iOS platforms, increasing at a rate of approximately 200 mHealth apps per day [1]. Available mHealth apps target a broad spectrum of health issues, such as bipolar disorder, cognitive impairment, cancer recovery, and substance use [2–5]. mHealth apps have the potential to collect a vast amount of data that could be used for researchers addressing disease prevention, early diagnosis, monitoring of chronic conditions, and the promotion of healthy habits and lifestyles amongst users. The potential benefits of mHealth include the ability to target large populations, access a wide variety of data sources in real-time, and the appeal of accessible and anonymous participation for users [6]. Research has also demonstrated the effectiveness of mHealth interventions, which may help to reduce barriers to treatment and empower consumers with more choice and control over their own health management [7].

Despite the potential benefits of mHealth, there are significant issues that impede their ability to become prescribable [8]. First, the vast number of mHealth apps available can make it difficult for users to identify and access evidence-based programs. While many mHealth apps include descriptions of the apps’ effectiveness, very few appear to have been subjected to robust evaluations [8, 9]. This limitation may not be apparent to consumers, who may assume that apps uploaded to app stores (such as Google Play for Android and the App Store for iOS) are routinely screened for clinical utility alongside testing for quality and malicious content [10, 11]. Second, the lack of systematic monitoring of mHealth apps has contributed to disproportionate targeting of health support to already advantaged groups, resulting in a bias against users who could benefit from mHealth support the most [12]. Third, data privacy and security concerns may leave users of mHealth apps at risk [13, 14]. Commercial mHealth apps are not required to adhere to the ethical data management guidelines mandatory in human research, which may leave users’ sensitive health data at-risk [15]. While accreditation programs have been introduced to bring standardisation in mHealth data privacy practices, evidence suggests that accredited apps still contain issues in systematically complying with data protection principles [16]. Combined, these issues highlight the need for improved, evidence-based design of mHealth apps to provide better quality support to users.

Health researchers are poised to develop and evaluate clinically useful mHealth tools, however they may be impeded by cost and technical skillset barriers [17]. The initial development costs of mHealth apps can range from the thousands to the hundreds of thousands of dollars, with factors such as the number of screens, complexity of content (static or dynamic), and inclusion of media files (e.g. video), all contributing to higher costs [18, 19]. Further, there are many non-trivial issues in the development and effective design of the components of mobile health systems currently being addressed by the mobile computing research community, such as energy efficiency, data management, human-computer interaction factors, and developing flexible and generic APIs to support health apps [20, 21]. While there have been efforts to establish tools for assessing the quality of health mobile apps, such as the Mobile App Rating Scale [22], non-experts from interdisciplinary fields wishing to deploy complex health interventions onto mobile devices may still face significant technical challenges. The initial cost and technical skillset barriers that arise in the app development process may reduce the quality of the deployed application, resulting in challenges for users in adopting the technology as an effective support for their wellbeing.

To help improve the quality of mHealth apps, health researchers and clinicians require access to low-cost and easy-to-use app development tools. Reducing the cost-to-entry for health experts can help to improve the quality of mHealth apps available by enabling researchers to iteratively evaluate and improve their systems. Commercial tools have been developed to address the skillset barrier that health researchers may experience in attempting to create and deploy mHealth apps, for example, by using a drag-and-drop builder interface. However, such tools typically have high access fees ($1000+ per year), are limited to Android or iOS devices only, and may not include components integral to the development of app-based interventions, such as video and audio components (for a review, see Connor, [23]). In contrast to commercial solutions, there also exist many powerful questionnaire platforms for social science, including QuestionSys [24–26] and PACO [27]. However, these platforms primarily focus on observational research methods such as health monitoring, without a strong focus on deploying health interventions. Therefore, there is a need for health researchers to have access to feature-rich tools for deploying both survey and intervention studies with low setup costs and minimal technical skills required.

In this paper, we introduce an open-source, distributed mobile research platform titled ‘*schema*’, which aims to incorporate the benefits of mHealth while overcoming the challenges and limitations mentioned above. *schema* provides a low technical skill method for deploying complex study designs onto both iOS and Android mobile devices, while allowing the researcher to store data on their own server. Further, the open-source nature of *schema* enables researchers to develop mHealth apps at minimal cost by building on the existing platform, while also allowing the research community to contribute new features. We begin by first describing the platform, including its architecture and primary features. We then detail several use cases for *schema* to illustrate its flexibility for supporting the various research and intervention designs used in mHealth studies. Finally, we conclude by discussing the strengths and limitations of the *schema* platform for research, and outline plans for future development and analysis.

## Implementation

*schema* is a mobile application developed for mHealth research. Researchers create a study protocol by editing a JavaScript Object Notation (JSON) file, which requires similar skills to editing syntax for analysis. This study protocol file contains a range of metadata, including measures, intervention content, and information for scheduling notifications. Further, researchers must also set up a web server to host the study protocol file and to receive and store data from *schema* using a text file or SQLite database. Participants can then enroll in the study by entering the study protocol URL into the *schema* app. We are currently hosting a public version of *schema* on the App Store and Google Play that can be used by researchers at no cost. Alternatively, researchers can download the source code from GitHub and deploy their own version of the app through the Apple Enterprise Developer Program for iOS, or by distributing the application package file directly to Android participants [28, 29].

There are several key strengths of the *schema* platform for health researchers. First, *schema* uses a distributed design whereby researchers host both their study protocol and participant data on their own server. This ensures that the creator of the study maintains complete ownership and responsibility for their study protocol and participant data. Second, *schema* is open-source, allowing the community to contribute new features to the application, or fork the development into a custom version that better suits the needs of a study design [28]. Thirdly, *schema* is built on a hybrid development platform, meaning that the app uses a single codebase for both the iOS and Android versions. This reduces the complexity and time required to implement new features into the app and ensures a consistent user experience for participants on both mobile platforms. While detailed deployment instructions and a sample JSON study protocol are provided in the GitHub repository, the following sections will provide a high-level overview of key features of the *schema* platform.

### Modules

The study protocol developed by a health researcher using *schema* consists of one or more modules. Modules appear as tasks on the home screen of the app for participants to complete. Each module consists of a series of elements split into sections that appear on different pages that communicate information to the participant (e.g. videos, audio, or instructional text) or require input from the participant (e.g. text input, sliders, and multiple-choice questions). These modules can be triggered to appear on a flexible schedule, as explained in the next section. Each module can also provide feedback to the participant via a dynamic graph (discussed below).

### Survey/intervention elements

Modules can be populated with a wide range of survey and intervention elements that are supported by *schema*, including sliders, date/time pickers, checkboxes, radio button groups, text input, labels, images, videos, and audio clips. Each element has a range of properties that need to be specified in the study protocol file, some of which are optional. For example, all elements require an *id* (a unique variable name within the task that is uploaded to the server with participant response data), *type* (the type of element it is, e.g. slider), and *text* (the question or instruction for this element). Basic HTML tags are supported within the *text* attribute for additional styling. Each element can also include optional properties that indicate whether a response is required, whether the element should be randomised, or to trigger branching logic. Elements in *schema* support branching logic whereby specific elements can be configured to become visible or hidden depending on the response provided to another element. Currently, elements that support this behaviour include the slider, Boolean (yes/no), checkbox, and radio button group elements. An element that is set to trigger by branching contains a reference to the element that will show or hide it and the value required to trigger branching. Finally, each individual element type has some unique attributes, e.g. a slider requires minimum and maximum values, whereas audio or video elements requires the URL of the relevant media. The complete list of supported elements and required properties are listed in detail on the *schema* GitHub repository [28].

### Feedback graphs

*schema* offers the ability to track responses to individual outcome variables and provide feedback to the participant via graphs. Each module can optionally specify a type of graph (bar or line), the ID of the element to graph (e.g. “q10”), graph title and description, and the maximum number of points to graph at once. Once the participant starts completing modules that have a dynamic feedback graph enabled, participants will be able to track their progress from within the app in the feedback screen. This feature is useful for allowing a participant to track their behavior change and progress through a study within *schema*.

### Flexible scheduling of modules and notifications

*schema* offers dynamic module presentation and notification schedules. When modules become available to complete, the module appears in the app’s home screen and the participant is alerted via a notification. Researchers can schedule notifications within the study protocol file to appear at a specific or random time and to display a custom message. Alternatively, modules can be set as permanently available from the home screen, enabling participants to access the module at any timepoint they wish. Table 1 details the different scheduling types supported by *schema*. As both Android and iOS enforce restrictions on the number of notifications single app can set at any one time, *schema* dynamically schedules the next 30 notifications every time the participant accesses the home screen of the application (e.g. when the app is opened or after a module is completed).

**Table 1:** Scheduling types supported by *schema*

Scheduling type	Description
Fixed	A notification appears when the module becomes available at a specified time. This module will remain available for the duration of the study, meaning that the participant can access it and respond at any time. Modules required from the beginning of a study can be scheduled to be available from the day prior to enrollment.
Interval	A notification appears when the module becomes available at a specified time or array of times. This schedule can be set to start on the day of enrollment or after a specified number of days, and set to repeat for a specified duration of days. Modules will remain in the task list and disappear after the participant responds.
Random	Similar behaviour to *Interval*, however each notification time is set randomly within a specified number of minutes relative to the notification time.
Timeout	Similar behaviour to *Interval*, however each module becomes unavailable to the user if not responded to within a specified number of minutes relative to the notification time.
Mixed	These scheduling types can be mixed, for example, a module can use both *Random* and *Timeout* rules to display notifications and make tasks available.

### Randomisation

*schema* supports several randomisation features*.* Modules can be scheduled within a random timeframe, as mentioned in the previous section on flexible notification scheduling. Using this feature, the order of the modules could be counterbalanced. For example, if a study protocol provides participants with a set number of intervention and feedback modules, they could all be set to randomly schedule within 1 h of each other. This means that each participant who enrolls in the study will receive the modules in a random order. Beyond counterbalancing the module delivery schedule, *schema* also supports counterbalancing within individual modules. First, *schema* can randomly shuffle the sections in a module every time a participant accesses it. Second, *schema* can randomly shuffle the questions within an individual section every time the participant accesses the module. This feature can be useful to overcome ordering effects in surveys and interventions.

Another randomisation feature supported by *schema* is random allocation of participants to study conditions upon enrollment. Each study protocol can specify a list of one or more conditions (e.g. control, intervention) in its metadata. Each module within the program can be allocated into a single-condition only or be made available for participants in all conditions. Alternatively, researchers cold host two study protocols on the same server with different file names, with researchers randomly allocating participants to conditions and providing separate study URLs accordingly.

Finally, specific elements within a module can be set to appear randomly every time a participant accesses the module. Researchers can achieve this by specifying that several elements belong to a group. When the participant opens the module, *schema* will select a random element from each group and display it to the participant, hiding the other group elements from display. This can be useful for randomly presenting different research conditions to an individual participant across the duration of a study protocol.

### Caching

Study media (i.e. video, audio or image) are hosted on the researcher’s server and embedded in each program via direct URLs. *schema* provides an option to have the media cached for offline use, with a recommendation to optimise media to reduce loading times. If this setting is enabled, *schema* will download all media elements when the participant first enrolls in the study and access the cached media whenever a module is accessed. This allows participants to progress in a study protocol, even when they do not have a stable internet connection. Response data are also cached offline whenever a module is completed. If a network connection is available after completing a module, *schema* will upload the data to the researcher’s server; otherwise, it will try again at a future point in time. The data includes the timestamp of when the participant responded, ensuring that the researcher has accurate information on the participant’s response patterns regardless of network issues.

### Localisation

As each research study that is deployed through *schema* loads study content via a JSON study protocol file, this content can be written in any language which will be displayed by the app. To translate any headings and instructions that are coded into the app itself, *schema* incorporates a translation library. Researchers must copy the English language assets file (a JSON file) and translate the strings of text into the desired language, and add this translated file back into the assets folder. The app will automatically detect the language setting on the device and load the correct language file. Further instructions are provided on the GitHub repository.

## Results

### Use cases of the *schema* platform

The following sections provide an overview of the intended use cases of *schema* for both participants and researchers who are deploying study protocols to participants.

#### *schema* for participants

**Fig. 1:**
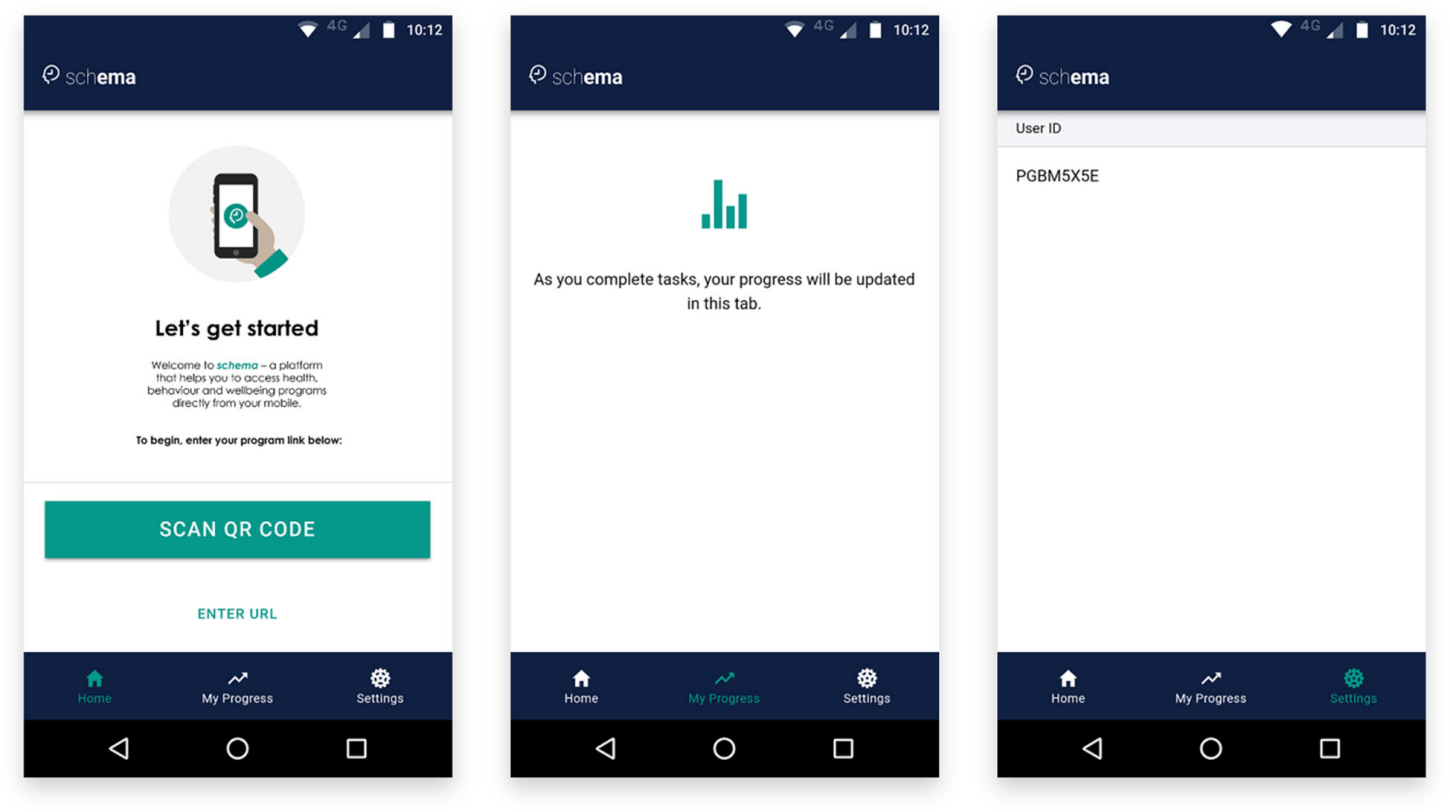
Screenshots of each of the tabs in their default state (left: Home tab, centre: My Progress tab, right: Settings tab, on an Android device)

Upon installing the app, participants have access to three primary tabs: *Home*, *My Progress*, and *Settings* (see Fig. 1). The *Home* tab invites the participant to scan a QR code or enter a URL directly to enroll in a study. Prior to enrolling, the *My Progress* tab displays a message that feedback graphs will appear after enrolling in a study and completing tasks, and the *Settings* tab displays a unique, anonymous, 8-digit user identifier. In research designs that require linking identifiers between different systems, participants can access their user identifier from the Settings tab and provide this to the investigators before enrolment.

**Fig. 2:**
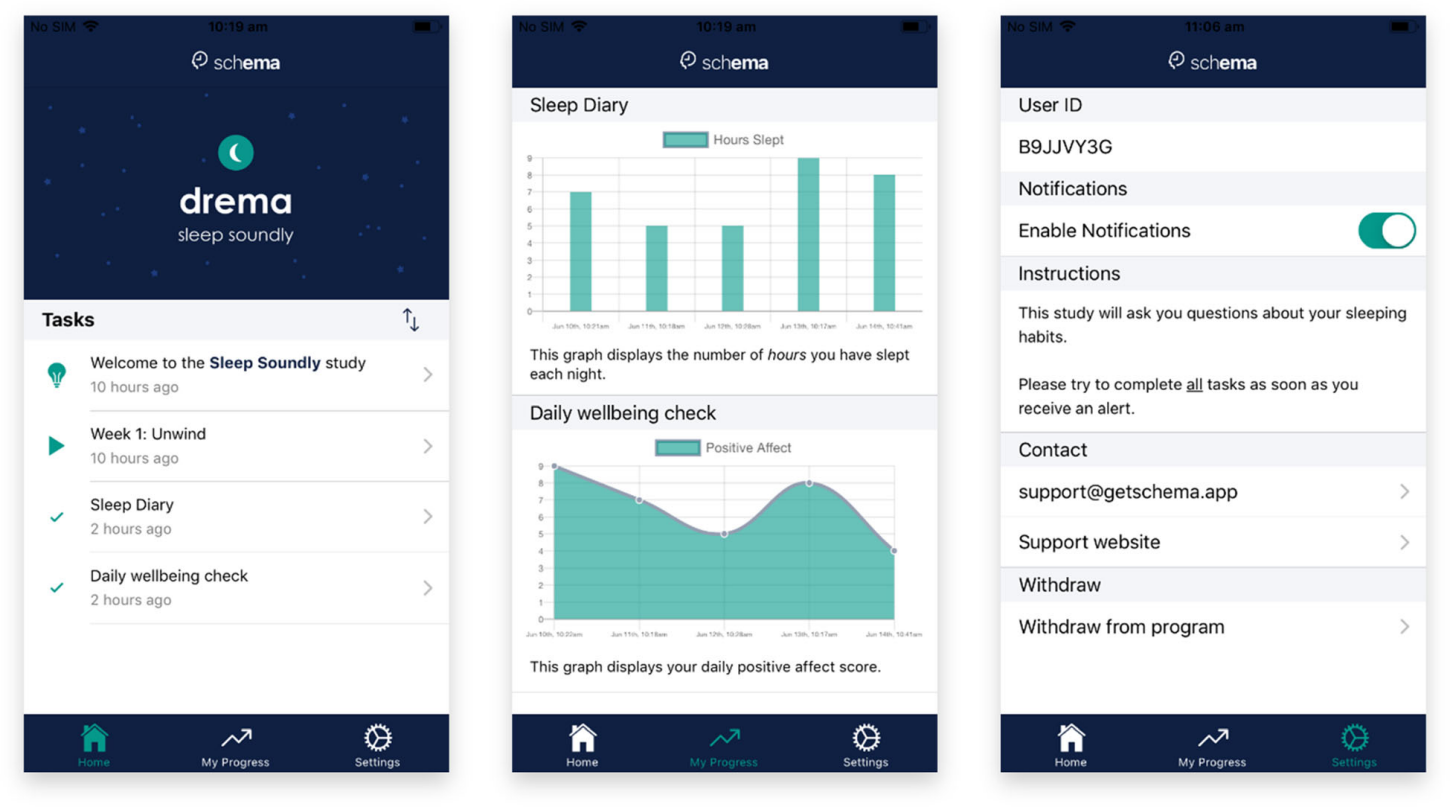
Screenshots of the three main tabs during an example study, ‘drema’ (on iOS). Left: Home screen displays the study banner and a list of available modules. Centre: Bar (top) and line (bottom) graphs display the participants’ responses to specified questions, dynamically updating as the participant progresses through the study protocol. Right: Details of the study protocol are displayed for the participant, including contact details of the research team

To enroll in a study, the participant selects one of the options from the *Home* tab. If the participant selects “Scan QR Code”, the device’s camera will open and attempt to detect a valid QR code and obtain its URL. If the participant selects “Enter URL”, a prompt will appear in which the participant directly types in the study URL. After scanning a QR code or entering a URL, the app attempts to download the contents of the study URL, and if it points to a valid *schema* protocol, it will download it and enroll the participant into the study. Figure 2 displays each of the tabs after a participant has enrolled in a valid study.

During a study, participants will be prompted to complete tasks by local notification. Clicking on a notification will open the home tab of the application, which will list all current tasks that the participant has not yet completed. Each task is represented by a type (e.g. information, video, audio, or survey), a title, and the time elapsed since the task became available (e.g. “21 minutes ago”). Clicking a button in the header will sort the task list by scheduled time (earliest to latest, or vice versa). The task list can be refreshed by dragging the list downwards until a loading spinner appears at the top of the screen.

**Fig. 3:**
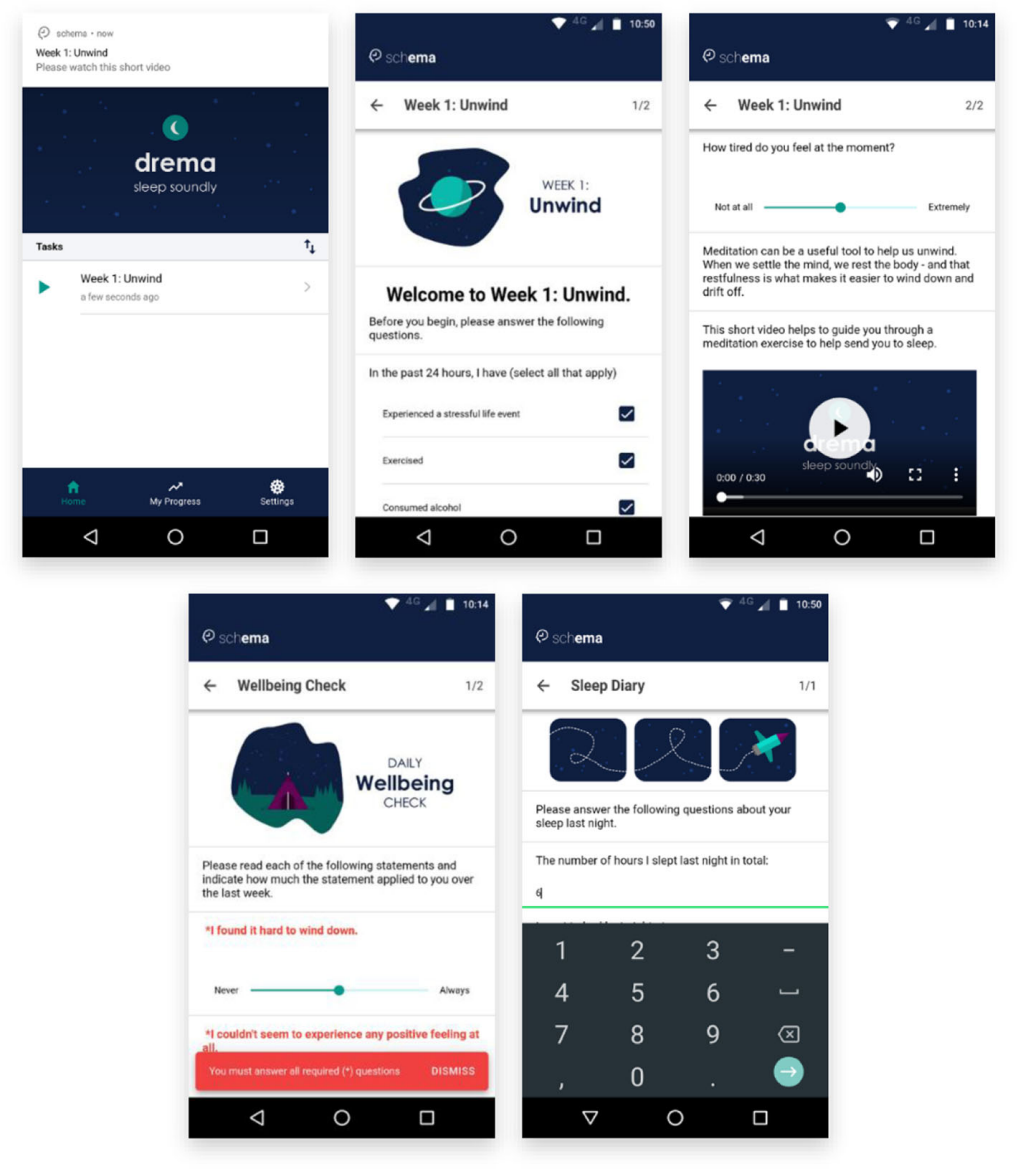
Screenshot of a module within drema titled “Week 1: Unwind”. Top left: The module is scheduled to appear on the home screen, and the participant is alerted via a notification. Top centre and top right: The module contains a range of elements, including images, instructional text, checkboxes, a slider, and a video file. Bottom left: Forced response elements will not allow participants to proceed without responding. Bottom right: A number element will only allow the participant to input a number as a response

After clicking on a task from the list, the app loads the elements for this task and navigates to a screen displaying these elements (see Fig. 3). Each task may be separated into sections containing one or more elements. Each element can present different input options to the participant, for example, a date element will display a date picker, whereas a text input could display a text-based or numeric keyboard. Once the participant has responded to all elements, they can proceed to the next section or complete the task by tapping the button at the bottom of the screen. If a participant has not responded to all required elements, a message will display and highlight the required questions. Otherwise, the response is saved, and the participant is navigated back to the home tab.

The *Settings* tab presents the participant with information and options regarding the current study (see Fig. 2), including the participant’s user ID, option to disable notifications, study description, researcher contact details (email and website), and an option to withdraw from the study protocol. Should a participant choose to withdraw from a study, *schema* will stop collecting data and any responses that have not yet been uploaded will be deleted (e.g. if the participant is offline). Importantly, researchers need to inform participants that they will need to contact the primary investigator directly if they wish to have any data that they have already contributed removed from the researcher’s database.

#### *schema* for researchers

The distributed nature of *schema* means that each researcher is responsible for setting up and maintaining the server that supports his or her study. Therefore, there are two primary considerations for researchers using *schema*: (1) creating, hosting and distributing a study protocol file, and (2) setting up a server to collect data. Detailed instructions for setting up a study protocol (including a sample protocol) and data collection server are provided on the *schema* GitHub page [28], therefore, this section will provide an overview of the process at a high level only.

##### Creating, hosting and distributing study protocol

**Fig. 4:**
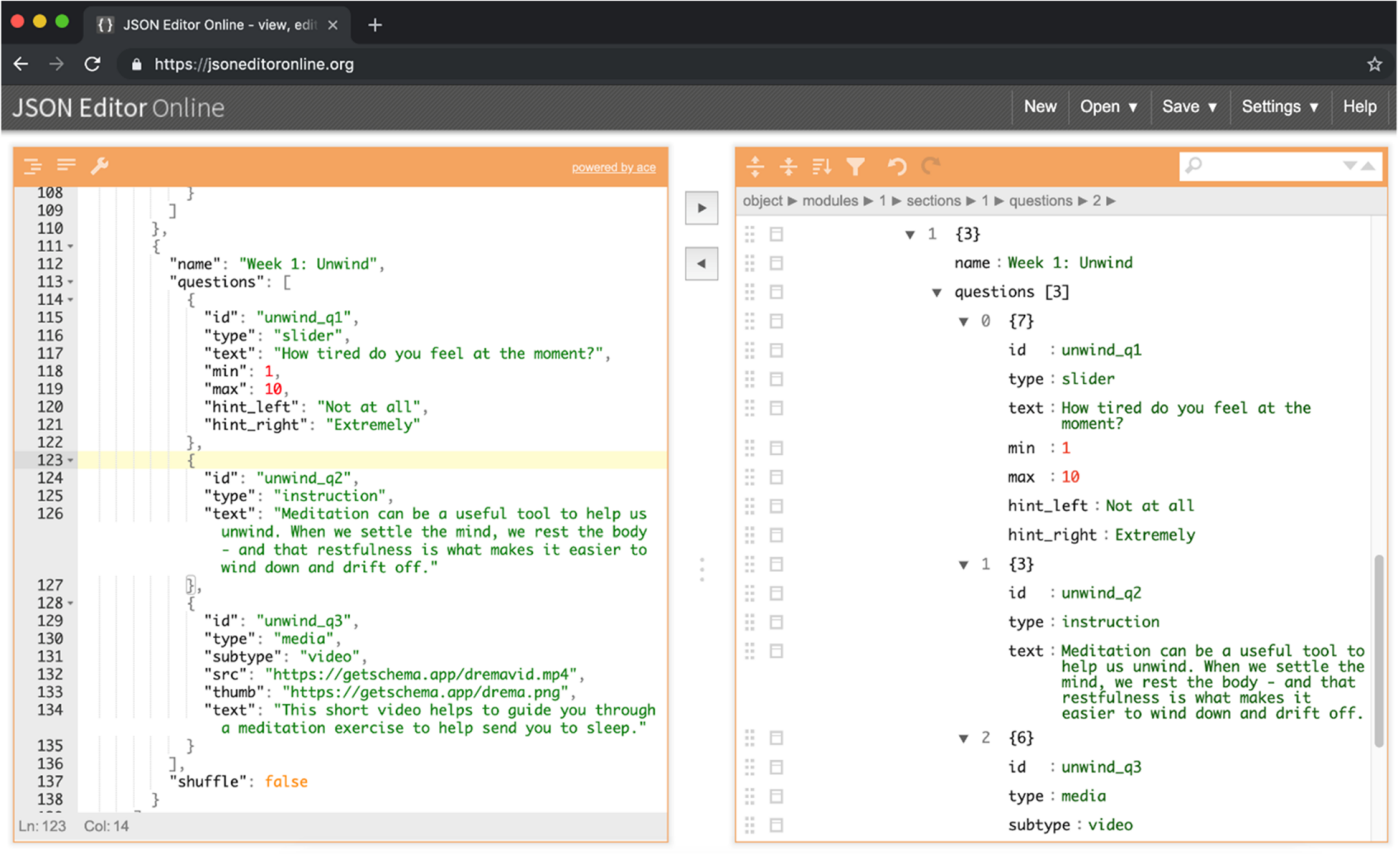
Researchers can create study protocols in JSON by editing the syntax directly or using a JSON editing tool

Study protocols are defined as JSON files – a language-independent data format in which human-readable, key-value pairs represent objects. Although editing JSON files requires a strict syntax, it does not require high-level technical skills and is therefore suitable for researchers who have experience with syntax for other applications, such as data analysis (see Fig. 4). The study protocol contains all the information required by *schema* to render a study and its tasks within the app, as well as schedule flexible notification and display dynamic feedback graphs. JSON files can be created in any plaintext editor (e.g. notepad) or by using a dedicated JSON editor (e.g. de Jong, [30]). Once the file has been created, it should be hosted on a public web server. Participants can then enroll in the study by entering the complete URL path to the JSON file into the app. We recommend shortening the URL using a service such as Bitly [31] or creating a QR code that points to the study protocol’s URL using an online service (e.g. QR Code Monkey, [32]).

##### Collecting data from the *schema* app

One of the required properties in the study protocol file is a URL where participant responses are submitted. Whenever a participant completes a task, *schema* will submit the response data to this URL as a POST request, including participant ID, task ID, timestamp, and responses to elements. This URL should point to a file on a server that can handle this data (e.g. a PHP file). Using the data within the POST request, the server can determine which study the data are related to which means that a single endpoint can be used for multiple studies. This also provides the researcher complete flexibility in how the data are stored, for example in a database or a text file. The exact variables that are submitted to the server are outlined on the GitHub repository [28], including user ID, module information, question responses, mobile operating system (i.e. Android or iOS), notification time and response time. While we provide a sample script on the *schema* GitHub repository for saving responses to a text file, we recommended that you seek support from a web developer for more complex data storage methods.

### Sample study protocols

The flexible and generic nature of *schema*’s study protocol allows support for a diverse range of study designs, including static survey (e.g. researcher report), cross-sectional surveys (observational), longitudinal surveys (observational), randomised-controlled trials (intervention), and just-in-time adaptive interventions (intervention), as discussed below.

#### Static survey

The simplest form of data collection that can be achieved using *schema* is a static survey in which the researcher inputs the data directly, for example, when collecting data in the field. Static surveys can be achieved by setting up a study with several pinned modules (e.g. those that allow multiple responses at any time without being hidden after completion) which the researcher completes as necessary. As all data are stored locally in the app and pushed to the server when an internet connection becomes available, *schema* is also suitable for fieldwork in remote areas with poor network coverage.

#### Cross-sectional surveys

Cross-sectional surveys can be deployed using a similar protocol as for static surveys; however, in this case, instead of the researcher inputting the data manually, participants enroll in the study using their own device which generates a unique user identifier. Each measure in the survey could be contained within a separate module, which is available on the home screen upon enrollment but disappears upon completion to ensures that the measure is only completed once by each participant. The survey link can be shared to participants who can then enroll in the survey after downloading *schema*. The advanced survey functionality could also support a variety of survey formats, e.g. a pre-survey with branching logic to screen for eligibility before continuing.

#### Longitudinal data collection

The app supports the design of short or long observational studies, including micro-longitudinal designs such as ecological momentary assessment (EMA), which aim to mitigate recall bias by capturing information from the participant in the moment [33]. Using the flexible notification and scheduling functionality of *schema*, modules can be scheduled to become available over time at fixed or random intervals. Further, the ability for notifications to timeout after a certain threshold has passed means that time-contingent data are captured within a specified period. Importantly for longitudinal studies, modules containing the same measure can be repeated over the study duration (e.g. daily reporting), enabling observation of trends over time. Longitudinal observation studies can be as complicated as necessary as *schema* supports the ability to use different notification schedules for each module.

#### Randomised controlled trials

*schema* can support randomised controlled trials in two ways. The study protocol can schedule surveys to appear in the home page and notify participants at fixed intervals: for example, a baseline survey upon enrolment in the study (time 1), a post-intervention assessment 3 months after enrolment (time 2), and a follow-up assessment 6 months after enrolment (time 3). There are two methods to randomly allocate participants to study arms. First, the design of study arms can be specified by setting a condition for each module, including the ability to specify a module to appear in all study conditions. Participants will then receive elements according to their condition protocol (e.g. audio and video). This method ensures double-blind randomisation, as both the researcher and participant have no input into the allocation of conditions. The second method for randomisation of participants is to develop multiple study protocols for a single study (one for each arm of the trial), which can be hosted on the same server under different file names. For this method, randomisation of participants would occur outside of the application, prior to providing participants with the instructions on how to download the app and enroll in the study. Such a method would be single-blind, as the research team would be allocating participants to conditions and distributing study protocol URLs accordingly.

The flexible notifications supported by *schema,* coupled with the randomisation techniques described, would also allow for more advanced study designs such as wait list controlled or within-subjects trials. For wait list controlled trials, participants can be randomly allocated using the above methods to either the intervention or wait list conditions. The wait list condition would consist of the baseline (time 1) and follow-up (time 2) surveys only, scheduled to appear at fixed time intervals, before the participants are able to access the intervention materials. For within-subjects trials, researchers can utilise *schema*’s functionality to randomise single elements from a set of distinct elements within a module. This allows individual participants to experience multiple conditions over repeated completions of a module.

Finally, the open source nature of *schema* allows researchers working on interventions that are more complicated to develop their own intervention elements and add them into the *schema* framework. At this stage, *schema* supports survey, image, audio, and video elements, which can be used to present intervention content. Interventions requiring elements that are more complex may use the existing platform as a baseline from which to develop custom elements, which will reduce development time and costs compared with a completely custom mobile application. Further, technically skilled members of the research community can contribute new features to be incorporated into the main *schema* codebase.

#### Just-in-time adaptive interventions

*schema* can also support a basic level of just-in-time adaptive interventions (JITAI) by using the branching functionality. JITAI are common mHealth research designs whereby pre-determined decision rules trigger the delivery of intervention modules [34]. Currently, *schema* can support simple JITAIs using the branching functionality. For example, a participant could be asked to answer a question about their current emotional state at several points throughout the day. Depending on the response provided by the participant, the module would display additional intervention content or elements; otherwise, this content would be skipped.

#### Micro-randomised trials

Finally, *schema* can also support micro-randomised trials through a combination of the branching and randomisation functionality. Micro-randomised trials involve the random assignment of an intervention when specific conditions are met, such as when the participant answers questions about their current context. In studies that use this design, intervention options may be randomly assigned hundreds of times, allowing researchers to determine which interventions are most effective at specific times, while also considering any moderating effects [35].

*schema* can support micro-randomisation using the notification, branching, and randomisation features discussed throughout this paper. For example, a participant could be alerted to a new survey module via a notification. Participant responses to the survey can trigger the presentation of an intervention module through branching logic. The intervention module could then utilise randomisation features, whereby the module may randomly present a control or intervention condition to the participant. The presentation of the initial survey module (and the subsequent branched intervention module) can then be repeated multiple times on fixed or random intervals, allowing the participant to a variety of intervention conditions at random over the duration of the study.

## Discussion

mHealth apps are a promising tool for health behaviour monitoring and intervention. However, cost and technical skillset barriers pose challenges for health researchers in developing evidence-based mHealth apps. In this paper, we presented *schema* - an open-source, hybrid, app-based platform for monitoring and intervention in mHealth research. *schema* incorporates a range of survey and intervention elements that can be delivered to participants with both iOS and Android devices. *schema* supports a range of functionality required to conduct robust evaluations of mHealth apps, including the ability to randomise content between or within participants, and flexible scheduling of content and notifications. Researchers can create study protocols using a human-readable syntax, and manage study data on their own secure server (e.g. a server managed by their institution). In so doing, *schema* significantly reduces the cost and technical skillset barriers that are common in mHealth app development. Low cost, open-source, feature-rich tools such as *schema* have important implications for improving the quality of mHealth apps available to the public through their ability to significantly reduce the impact of cost and technical skillset barriers to robust evaluations.

### Strengths and limitations

Importantly, *schema* has several strengths and limitations. First, *schema* uses a simple but flexible method for researchers to create and deploy studies and interventions to participants. *schema* can deliver many diverse study designs to participants through a simple, human-readable JSON file that can be created by researchers who have little experience developing app-based interventions. Second, *schema* supports a wide range of elements to allow for a diverse range of data collection and delivery of media to participants. Further, these elements support branching logic and randomisation that can add additional functionality and complexity to research studies, including novel study designs such as micro-randomisation. Third, the distributed nature of the platform ensures researchers maintain complete control of their study protocol and participant data, minimising the common concern of mHealth users surrounding the security and privacy of their sensitive health data. Finally, the open-source nature of the platform means that technically skilled researchers can also modify the project for their own needs or contribute to the further development of the project to benefit future research.

There are several limitations of the current *schema* platform that could be addressed in future work, either by the study authors or by the broader research community. First, there are many opportunities to implement personalisation and context-awareness within *schema* using artificial intelligence techniques. While *schema* supports a basic level of JITAI using branching logic, future development will explore an open-source, server-side platform to tailor programs to the individual user by performing calculations on the data and pushing the results back to the app. Personalisation of mHealth tools is a growing research area [36], however careful consideration is required to ensure that automated mHealth tools are ethical and effective for vulnerable populations.

Second, while *schema* provides a simple method for creating and deploying study protocols, there is scope to build additional tools that further simplify the development of study protocols. While we recommend that researchers using *schema* utilise existing JSON editor tools (e.g. de Jong, [30]), there is also scope for custom tools to be developed specifically for the purpose of creating study protocols for *schema*. It may also be beneficial to explore methods within such custom tools that would allow researchers to share modules that may be of use to other researchers, such as common measures and templates, further contributing to open science.

There is also significant scope to expand on the data collected by *schema*, particularly by incorporating data collection from mobile phone sensors. Mobile phones are typically equipped with a wide array of sensors that can provide data on the device’s position and environment. Mobile sensors could provide benefits for both researchers and participants of mobile apps. For example, sensor data such as the user’s current location can provide additional context to survey responses. Further, sensor data could also be used to personalise the application experience, delivering relevant content to the participant based on their current context. The Ionic platform provides a range of plugins that can incorporate native functionality like sensors into hybrid apps, including geolocation, device motion, and magnetometer [37]. We plan to explore this area further, as well as rely on the expertise of the open source community to add sensor-based features into the *schema* platform.

Finally, we also plan to test the effectiveness of *schema* with different clinical and research groups to further improve the usability and accessibility of the platform. While the platform may not provide as many features as a completely custom app, it does provide a low-cost method for deploying and evaluating monitoring and intervention programs. Researchers are encouraged to use *schema* to conduct pilot studies to determine the effectiveness of intervention content before investing more resources in the development of stand-alone apps for specific interventions. Developers can also build upon the open source code to develop and release stand-alone interventions. We are currently exploring the effectiveness of *schema* in several domains, including eating disorders, substance use research, and perinatal health, and we encourage other researchers to share data on the effectiveness of the platform in their own domains.

## Conclusions

To conclude, this paper presented *schema*, an open-source, distributed, app-based platform for researchers to deploy health behaviour monitoring and intervention apps. The combination of survey and intervention elements alongside dynamic scheduling and randomisation functionality can assist researchers to design and evaluate quality mHealth apps for medical research. Importantly, the open-source nature of *schema* reduces many of the barriers faced by researchers in developing and deploying mHealth apps. To get started with *schema*, please visit the GitHub repository [28].

## Availability and requirements

**Project name:** schema.

**Project home page:** https://github.com/schema-app/schema

**Operating system(s):** Android and iOS.

**Programming language:** TypeScript, HTML, CSS.

**Other requirements:** Ionic 4+.

**License:** MIT.

**Any restrictions to use by non-academics:** N/A.

## Data Availability

The *schema* platform is available as an open-source tool for researchers. The platform can be accessed from the GitHub repository (Shatte & Teague, [27]).

## Abbreviations

API: Application Programming Interface
App: Application
EMA: Ecological Momentary Assessment
HTML: Hypertext Markup Language
ID: Identification
iOS: Internetwork Operating System
JITAI: Just-in-time adaptive interventions
JSON: JavaScript Object Notation
mHealth: Mobile applications for health
QR code: Quick Response Code
SQLite: Structured Query Language Lite
URL: Uniform Resource Locator

## References

[1] Aitken M, Clancy B, Nass D (2017). The Growing Value of Digital Health: Evidence and Impact on Human Health and the Healthcare System.

[2] Nicholas J, Larsen ME, Proudfoot J, Christensen H (2015). Mobile apps for bipolar disorder: a systematic review of features and content quality. J Med Internet Res.

[3] Bateman DR, Srinivas B, Emmett TW, Schleyer TK, Holden RJ, Hendrie HC (2017). Categorizing health outcomes and efficacy of mHealth apps for persons with cognitive impairment: a systematic review. J Med Internet Res.

[4] Seiler A, Klaas V, Tröster G, Fagundes CP (2017). eHealth and mHealth interventions in the treatment of fatigued cancer survivors: a systematic review and meta-analysis. Psychooncology..

[5] Kazemi DM, Borsari B, Levine MJ, Li S, Lamberson KA, Matta LA (2017). A systematic review of the mHealth interventions to prevent alcohol and substance abuse. J Health Commun.

[6] Munos B, Baker PC, Bot BM, Crouthamel M, de Vries G, Ferguson I (2016). Mobile health: the power of Wearables, sensors, and apps to transform clinical trials. Ann N Y Acad Sci.

[7] Berry N, Lobban F, Emsley R, Bucci S (2016). Acceptability of interventions delivered online and through mobile phones for people who experience severe mental health problems: a systematic review. J Med Internet Res.

[8] Byambasuren O, Sanders S, Beller E, Glasziou P (2018). Prescribable mHealth apps identified from an overview of systematic reviews. npj Digit Med.

[9] Larsen ME, Huckvale K, Nicholas J, Torous J, Birrell L, Li E (2019). Using science to sell apps: Evaluation of mental health app store quality claims. npj Digit Med.

[10] Apple. App Review. 2019. Available from: https://developer.apple.com/app-store/review/ Archived at: http://archive.fo/elYM6. [cited 2019 Jun 11].

[11] Google Play. Developer Policy Centre. 2019. Available from: https://play.google.com/about/developer-content-policy/#!?modal_active=none Archived at: http://archive.fo/K7G0A. [cited 2019 Jun 11].

[12] Singh K, Drouin K, Newmark LP, Lee J, Faxvaag A, Rozenblum R (2016). Many mobile health apps target high-need, high-cost populations, but gaps remain. Health Aff.

[13] Dehling T, Gao F, Schneider S, Sunyaev A (2015). Exploring the far side of mobile health: information security and privacy of mobile health apps on iOS and android. JMIR mHealth uHealth.

[14] Khalid H, Shihab E, Nagappan M, Hassan AE (2014). What do mobile app users complain about?. IEEE Softw.

[15] Glenn T, Monteith S (2014). Privacy in the digital world: medical and health data outside of HIPAA protections. Curr Psychiatry Rep.

[16] Huckvale K, Prieto JT, Tilney M, Benghozi P-J, Car J (2015). Unaddressed privacy risks in accredited health and wellness apps: a cross-sectional systematic assessment. BMC Med.

[17] Gagnon M-P, Ngangue P, Payne-Gagnon J, Desmartis M (2016). M-health adoption by healthcare professionals: a systematic review. J Am Med Informatics Assoc.

[18] Nitze A, Schmietendorf A, Dumke R. An analogy-based effort estimation approach for mobile application development projects. Jt Conf Int Work Softw Meas Int Conf Softw Process Prod Meas. 2014:99–103.

[19] Altaleb AR, Gravell AM (2018). Effort estimation across Mobile app platforms using agile processes: a systematic literature review. J Softw.

[20] Joorabchi ME, Mesbah A, Kruchten P. Real challenges in mobile app development. ACM/IEEE Int Symp Empir Softw Eng Meas. 2013:15–24.

[21] Pryss R, Schobel J, Reichert M. Requirements for a Flexible and Generic API Enabling Mobile Crowdsensing mHealth Applications. In: 2018 4th international workshop on requirements engineering for self-adaptive, collaborative, and cyber physical systems (RESACS). IEEE; 2018. p. 24–31. Available from: https://ieeexplore.ieee.org/document/8501476/.

[22] Stoyanov SR, Hides L, Kavanagh DJ, Zelenko O, Tjondronegoro D, Mani M (2015). Mobile App Rating Scale: A New Tool for Assessing the Quality of Health Mobile Apps. JMIR mHealth uHealth.

[23] Connor TS. Experience sampling and ecological momentary assessment with mobile phones 2015. Available from: https://www.otago.ac.nz/psychology/otago047475.pdf.

[24] Schobel J, Probst T, Reichert M, Schickler M, Pryss R (2019). Enabling Sophisticated Lifecycle Support for Mobile Healthcare Data Collection Applications. IEEE Access.

[25] Schobel J, Pryss R, Probst T, Schlee W, Schickler M, Reichert M (2018). Learnability of a Configurator Empowering End Users to Create Mobile Data Collection Instruments: Usability Study. JMIR mHealth uHealth.

[26] Schobel J, Pryss R, Schickler M, Ruf-Leuschner M, Elbert T, Reichert M (2016). End-User Programming of Mobile Services: Empowering Domain Experts to Implement Mobile Data Collection Applications. 2016 IEEE international conference on Mobile services (MS). IEEE.

[27] PACO (2020). PACO: The Personal Analytics Companion.

[28] Shatte ABR, Teague SJ (2019). schema.

[29] Apple (2019). Apple Enterprise Developer Program.

[30] de Jong J. JSON Editor Online [Internet]. 2019. Available from: https://jsoneditoronline.org/ Archived at: http://archive.fo/OuSaP. [cited 2019 Jun 11].

[31] Bitly. bitly [Internet]. 2019. Available from: https://bitly.com/ Archived at: http://archive.fo/00Oi9. [cited 2019 Jun 11].

[32] QR Code Monkey. QR Code Monkey [Internet]. 2019 . Available from: https://www.qrcode-monkey.com/ Archived at: http://archive.fo/4SJhz. [cited 2019 Jun 11].

[33] Shiffman S, Stone AA, Hufford MR. Ecological momentary assessment. Annu Rev Clin Psychol. 2008;4:1–32. 10.1146/annurev.clinpsy.3.022806.091415 18509902

[34] Intille SS (2004). Ubiquitous computing technology for just-in-time motivation of behavior change. Stud Health Technol Inform.

[35] Klasnja P, Hekler EB, Shiffman S, Boruvka A, Almirall D, Tewari A (2015). Microrandomized trials: an experimental design for developing just-in-time adaptive interventions. Health Psychol.

[36] Shatte ABR, Hutchinson DM, Teague SJ. Machine learning in mental health: a scoping review of methods and applications. Psychol Med. 2019:1–23. 10.1017/S0033291719000151 30744717

[37] Ionic. Community Plugins [Internet]. 2020. Available from: https://ionicframework.com/docs/native/community. [cited 2020 Mar 16].

